# Salinity Effect in Permeability of Salt in Nanofiltration and Reverse Osmosis Membranes

**DOI:** 10.1002/open.202500198

**Published:** 2025-11-14

**Authors:** A. Lachheb, A. El Attar, F. Z. Addar, I. Kouda, N. Zouhri, J. Touir, M. Taky, M. Tahaikt

**Affiliations:** ^1^ Laboratory of Advanced Materials and Process Engineering Faculty of Sciences Ibn Tofail University Kenitra 1246 Morocco; ^2^ École Normale Supérieure Moulay Ismail University Meknes Morocco; ^3^ International Water Research Institute. Mohammed VI Polytechnic University Lot 660, Hay Moulay Rachid Ben Guerir 43150 Morocco

**Keywords:** desalination, modeling, nanofiltration, reverse osmosis, transfer mechanism

## Abstract

In Morocco, water resources are increasingly under threat due to population growth, economic expansion, and climate change. Among the proposed solutions, brackish water desalination using membrane technologies such as nanofiltration (NF) and reverse osmosis (RO) with low‐pressure membranes presents a promising alternative. This study evaluated the impact of salinity on the performance of two nanofiltration membranes (NF270 and NF90) and one reverse osmosis membrane (TM710) using three semisynthetic brackish water samples with salinities of 2, 4, and 6 g L^−1^. Ion transfer mechanisms, particularly for sodium (Na^+^) and chloride (Cl^−^), were analyzed using the Spiegler–Kedem (SK) and Kedem–Katchalsky (KK) mathematical models. Additionally, the effects of salinity on diffusion flux (Jdiff), convection‐induced concentration (Cconv), reflection coefficient (*σ*), and solute permeability (Ps) were examined. Results indicate that the NF270 membrane exhibits the highest permeate flux, while NF90 and TM710 perform similarly. For all three membranes, permeate flux decreases almost linearly as feed water salinity increases. Regarding total dissolved solids (TDS) rejection, the TM710 membrane achieves the highest removal efficiency, followed by NF90 and then NF270. The NF270 membrane shows greater convective transport than NF90, with both diffusive and convective fluxes increasing with salinity. In contrast, the TM710 membrane operates primarily through diffusion, with TDS having little effect on its diffusion flux. NF90 and TM710 exhibit similar *σ* and Ps values for sodium and chloride ions, independent of TDS, highlighting the NF90's similarity to a reverse osmosis membrane. In contrast, for NF270, the sodium reflection coefficient (*σ*) increases with TDS, while solute permeability (Ps) rises for both ions due to a decline in retention efficiency.

## Graphical abstract

This study assesses the effect of feed salinity (2–6 g L^−1^) on the performance of two nanofiltration membranes (NF270, NF90) and one reverse osmosis membrane (TM710). NF270 provides the highest permeate flux, while TM710 achieves the best TDS rejection. Increasing salinity reduces flux for all membranes. The Spiegler–Kedem and Kedem–Katchalsky models describe ion transport mechanisms for Na^+^ and Cl^−^.

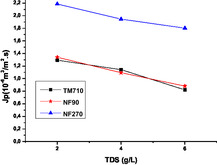

## 1 Introduction

Water scarcity and the safety of drinking water are among the most pressing challenges for sustainable socioeconomic development, particularly in regions with limited freshwater resources.^[^ ^1^ ^]^ Currently, over 2 billion people live in countries experiencing severe water stress,^[^ ^2^ ^]^ a situation expected to worsen in the coming decades. By 2050, global freshwater withdrawals are projected to increase by 55%, potentially exposing nearly 40% of the world's population to critical water stress conditions.^[^ ^3^ ^,^ ^4^ ^]^ Beyond the challenge of water availability, contamination by pathogens and chemical pollutants poses a significant public health risk.^[^ ^5^ ^]^ Brackish water, for instance, typically has a salinity ranging from 1 to 10 g L^−1^^[^ ^6^ ^]^ and is often consumed in areas where freshwater is scarce. However, prolonged consumption has been linked to various health issues, including digestive disorders, skin infections, hypertension, kidney stones, and, in some cases, an increased risk of certain cancers.^[^ ^1^ ^]^

Faced with these challenges, ensuring a sustainable and secure water supply requires the development of efficient treatment technologies. Among the available solutions, desalination has emerged as a key approach to guaranteeing access to drinking water, particularly in high‐salinity regions.^[^ ^7^ ^,^ ^8^ ^]^ This process is generally divided into two main techniques: thermal desalination and membrane‐based desalination.^[^ ^9^ ^]^ In recent years, membrane technologies such as nanofiltration (NF) and reverse osmosis (RO) have gained prominence due to their ability to treat seawater and brackish water (BW), enabling the production of drinking water, wastewater reuse, and the purification of industrial and municipal effluents.^[^ ^10^, ^11^ ^–^ ^12^ ^]^

In Morocco, the water management strategy focuses on brackish water desalination to diversify resources and ensure a sustainable supply despite climate fluctuations and growing demand.^[^ ^13^ ^]^ The desalination of BW has garnered significant attention due to its lower energy consumption compared to seawater desalination.^[^ ^14^ ^]^ BW, which typically has a salinity of 1–10 g L^−1^, is often found in inland aquifers and coastal areas where freshwater resources are over‐exploited. The desalination of BW is not only more energy‐efficient but also less costly than seawater desalination, making it a more viable option for regions that face a moderate salinity level in their water sources. This makes the desalination of BW an attractive solution for Morocco and other countries with similar challenges, as it offers a balance between energy consumption, cost‐effectiveness, and water quality.

Several research projects have been conducted in various regions of the Kingdom of Morocco, such as Zagora, M’rirt, and Khénifra, to explore desalination technologies for brackish water treatment. These studies are particularly relevant in the context of increasing water scarcity, where optimizing desalination technologies is essential to ensuring sustainable access to drinking water.^[^ ^15^, ^16^ ^–^ ^17^ ^]^

The choice of desalination technology largely depends on the characteristics of the water to be treated, particularly its salinity and ionic composition. In this regard, NF and RO membranes play a central role in providing solutions tailored to different salinity levels and various water quality requirements.

RO membranes feature exceptionally small pores, typically in the range of a few angstroms (Å),^[^ ^18^ ^]^ enabling ultra‐fine filtration. This allows them to effectively reject a wide range of ions and molecules, making RO highly efficient for seawater desalination and the treatment of low‐salinity brackish water. In contrast, NF membranes possess slightly larger pores, ranging from 0.1 to 1 nm,^[^ ^19^ ^]^ which imparts distinct separation characteristics. They are capable of retaining a significant portion of divalent ions, such as calcium (Ca^2+^) and magnesium (Mg^2+^), while permitting the easier passage of smaller monovalent ions like sodium (Na^+^) and chloride (Cl^−^). This selective permeability is further defined by the nominal molecular weight cut‐off (MWCO) of NF membranes, typically between 100 and 5000 Da,^[^ ^20^ ^]^ emphasizing their ability to strike a balance between ion rejection and permeability.

Following the advent of low‐pressure membranes tailored for BW treatment, the transport mechanisms within NF membranes have emerged as notably intricate. Unlike RO membranes, which predominantly rely on solubilization and diffusion driven by concentration gradients and osmotic pressure, NF membranes function through a more complex interplay of mechanisms. These membranes operate primarily via pressure‐driven convection and solubilization‐diffusion, which collectively govern the selective transport of solutes across the membrane, leading to distinct separation characteristics.^[^ ^21^, ^23^ ^–^ ^24^ ^]^ The separation efficiency of NF systems, in particular, is governed by a complex interaction of steric, dielectric, and electrostatic partitioning effects that occur between the membrane and the solution.^[^ ^25^, ^26^ ^–^ ^27^ ^]^ These factors depend not only on the membrane material itself but also on the feed composition, pH, temperature, and other operating conditions.^[^ ^28^ ^]^

To enhance the understanding and prediction of the performance of both NF and RO membranes, mathematical models such as the Spiegler–Kedem (SK) and Kedem–Katchalsky (KK) models are widely used. These models offer valuable insights into the complex interactions that govern solute transport, permeability, and rejection characteristics under varying operational conditions. The KK model is grounded in thermodynamic principles, offering a comprehensive description of membrane selective permeability by accounting for osmotic and hydraulic pressure differences and the interactions between solutes and the membrane. In contrast, the SK model primarily focuses on the relationship between osmotic pressure, hydraulic pressure, and membrane properties. While the KK model provides a more detailed thermodynamic analysis, both models are crucial for predicting flux behavior and rejection rates.^[^ ^27^ ^,^ ^29^ ^,^ ^30^ ^]^

This study has two main objectives. Firstis to evaluate the performance of two NF membranes (NF270 and NF90) and one RO membrane (TM710) in the partial demineralization of three semisynthetic brackish water samples with salinities of 2, 4, and 6 g L^−1^, by examining the impact of operational parameters, such as TMP applied and initial feed water salinity, on the retention of Na^+^ and Cl^−^ ions. Secondly, the study uses the SK and KK models to investigate the influence of salinity on the phenomological coefficients of these two models.

## 2 Experimental Section

### 2.1 Unit Pilot

**Figure 1:**
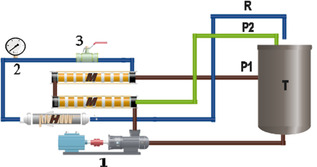
Schematic diagram of the NF/RO pilot plant. T: tank; M: nanofiltration module; P: permeate recirculation; R: retentate recirculation; H: heat exchanger; 1: high‐pressure pump; 2: pressure sensor; 3: pressure regulation valves.

The experiments were conducted on a semiindustrial pilot, provided by the French company TIA. It consists of a feed tank with a capacity of 150L, a pump (P) model CAT PUMP which offers a pressure up to 100 bars. It is equipped with two spiral modules in series. The retentate from the first module is admitted into the second module. The pressure drop is 2 bars, one bar per module (**Figure** **1**).^[^ ^31^ ^]^

For each applied pressure value, we measured the following parameters: conversion rate (%Y), the retention rate (%R), and permeate flux (J_v_).

The permeate flux is given by the equation^[^ ^32^ ^,^ ^33^ ^]^

(1) $$J_{v}=\frac{Q_{p}}{S}\left(\frac{L}{m^{2}.s}or\frac{m^{3}}{m^{2}.s}\right)$$

where S is the membrane surface area (*m* ^2^), and $Q_{P}$ is the flow rate of the permeate (L h^−1^ or m^3 ^s^−1^).

The conversion rate (Y) is defined as

(2) $$Y\left(\%\right)=\frac{Q_{p}}{Q_{0}}\times 100$$

where $Q_{P}$ is the permeate flow (L/h), and $Q_{0}$ is the feed flow (L/h).

Retention rate (R) is defined as

(3) $$R=\left(1-\frac{C_{P}}{C_{0}}\right)\times 100$$

where *C_p_* is the solute concentration in permeate (g L^−1^), and C_0_ is the solute concentration in the feed water (g L^−1^).

The permeability of membranes is an intrinsic characteristic of the membrane that depends on its structure. It is closely related to the temperature of the raw water.^[^ ^34^ ^]^ Permeability ($L_{p}$) can be practically defined as the ratio of the permeation flux ($J_{v}$) to the effective transmembrane pressure ($\text{Δ}P_{M}$)

(4) $$L_{p}=\frac{J_{v}}{\text{Δ}P_{M}}$$

Substituting the permeation flux ($J_{v}$) into (Equation 4), we obtain

(5) $$L_{p}=\frac{Q_{p}}{\text{Δ}P_{M}}*\frac{1}{S}$$

In practice, the permeability of a membrane is determined as the slope of the line ($J_{v}$) versus ($\text{Δ}P_{M})$.

### 2.2 Membranes Used

The three membranes used in this study were supplied by Dow FilmTec (Edina, MN), including two NF membranes (NF90 and NF270) and one RO membrane for brackish water (TM710). The specifications of the membranes used are provided in **Table** **1**.

**Table 1:** Membranes used characteristics.

Membranes	NF904040	NF270 4040	TM710
Pressure maximum (bar)	41	41	41
Thin film layer	Polyamide	Polyamide	Polyamide
MWCO (Da, 30 °C)	200^[^ ^41^ ^]^	300^[^ ^41^ ^]^	<200
Area active (m^2^)	7.6	7.6	7
Tolerated pH during treatment	3 à 10	3 à 10	2 à 11
Maximum allowed temperature (°C)	45	45	45
Admitted level of free chlorine (ppm)	0.1	0.1	–
Silt Density Index (SDI)	<5	<5	<5

The experiments are carried out at room temperature (25 °C ± 2 °C). After experiments, the membranes are cleaned with alkaline and acidic cleaning solutions according to the manufacturer's recommendations.^[^ ^35^ ^]^

### 2.3 Physico‐Chemical Characteristics of the Feed Water

The three semisynthetic brackish water samples were prepared by doping groundwater with a conductivity ranging from 700 to 800 µS cm^−1^ (600 ppm), incorporating the following mineral salts: NaCl, CaSO_4_, CaCO_3_, and MgCl_2_, to achieve three salinities (2, 4, and 6 g L^−1^). The physicochemical characteristics of the three feed samples are summarized in **Table** **2**. Permeate samples are collected, and water parameters are analytically determined using the standard methods previously described.^[^ ^22^ ^,^ ^36^ ^]^

**Table 2:** Physico‐chemical characteristics of the feed water.

TDS [g L^−1^]	≈2 [g L^−1^]	≈4 [g L^−1^]	≈6 [g L^−1^]	Guidelines (OMS)
pH	7,2	7,4	7,5	6.5–85
Turbidity (NTU)	<1	<1	<1	<5
TDS [mg L^−1^]	1970	4030	5980	<1000
Ca^2+^ [mg L^−1^]	230	242	248	<270
Mg^2+^ [mg L^−1^]	78	91	95	<50
Na^+^ [mg L^−1^]	332	1120	1920	<200
HCO_3_^−^ [mg L^−1^]	305	313	310	–
SO_4_^2−^ [mg L^−1^]	311	320	317	<200
Cl^−^ [mg L^−1^]	574	1780	2980	<250

Those feed waters are characterized by high hardness and high sulfate and bicarbonate contents. it is very rich in chloride and sodium ions and that their mass concentrations largely exceed the standards.

## 3 Mathematical Models: Spiegler–Kedem (SK) and Kedem–Katchalsky (KK)

To determine the diffusive and convective transfer mechanisms, the SK and KK models have been used. These models describe transport phenomena through NF membranes based on the principles of diffusion and convection. The SK model is particularly relevant, suggesting that solute flux ($J_{s}$) is a combination of diffusive and convective fluxes, with the solute flux depending on membrane permeability ($L_{p}$) and transmembrane pressure TMP ($\text{Δ}P_{M}$) (Equation 6 and 7). Local transport coefficients introduced by the SK model allow for the determination of the overall flux ($J_{v}$) through the membrane, linking it to pressure and concentration gradients (Equation 8).

(6) $$J_{s}=J_{\text{diff}}+J_{p}.C_{\text{conv}}$$

(7) $$J_{s}=C_{P}.J_{p}$$

(8) $$J_{v}=-L_{p}(\frac{dp}{dx}-\sigma \frac{d\pi }{dx})$$

Additionally, solute rejection is described by (Equation 9), where rejection is related to the reflection coefficient (*σ*) and solute concentration within the membrane.

(9) $$R=1-\frac{C_{p}}{C_{f}}=\frac{\sigma (1-F)}{1-\text{σ}F}$$

These models provide a comprehensive description of solute and solvent transport across NF membranes, as extensively discussed in previous studies.^[^ ^22^ ^,^ ^37^ ^]^ They are crucial for predicting membrane filtration performance, with key assumptions including the use of pressure and concentration gradients as driving forces, and the independence of solute type, charge, solvent, and membrane properties. For further details, please refer to the previous studies.^[^ ^22^ ^,^ ^37^ ^]^

## 4 Results and Discussions

### 4.1 Effect of TMP on the Permeate Flux

**Figure 2:**
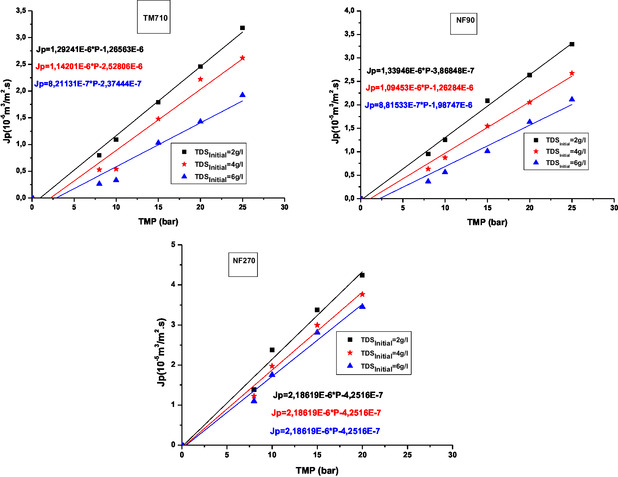
Experimental water flux through membranes vs. the TMP at different salinity.

**Figure** **2** shows, for the tested membranes and for different feed water salinities, the variation of the average flux of permeate as a function of applied TMP.

As shown in Figure 2, the permeate flux increases almost linearly with increasing operating pressure for the three membranes tested in the three feed TDS.

The NF270 membrane has the highest values of permeate flux. The NF90 membrane has slightly higher permeate flux compared to TM710. This is primarily due to the fact that TM710 is dense, whereas NF membranes are porous, even if NF90 shows a structure properties close to that of RO membranes.^[^ ^38^ ^]^ In addition, the pores of NF270 are larger than those of NF90^[^ ^31^ ^]^, The almost linear evolution of permeate flux with the TMP for the three membranes at the feed TDS studied shows that Darcy's law is valid.^[^ ^38^ ^]^

**Figure 3:**
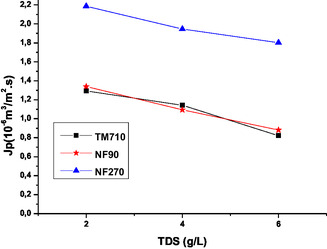
Variation of the permeate flux as a function of feed TDS.

**Figure** **3** shows, for the membranes tested, the variation of the permeate flux (J_p_) as a function of feed TDS.

The analysis of the results in Figure 3 shows that the permeate flux decreases almost linearly with the increase in feed TDS due to concentration polarization phenomena.^[^ ^39^ ^]^ The NF270 membrane exhibits the highest permeate flux values, while the NF90 and TM710 membranes show similar behavior with no significant differences.

### 4.2 Effects of TMP and Feed TDS on the TDS Permeate

**Figure 4:**
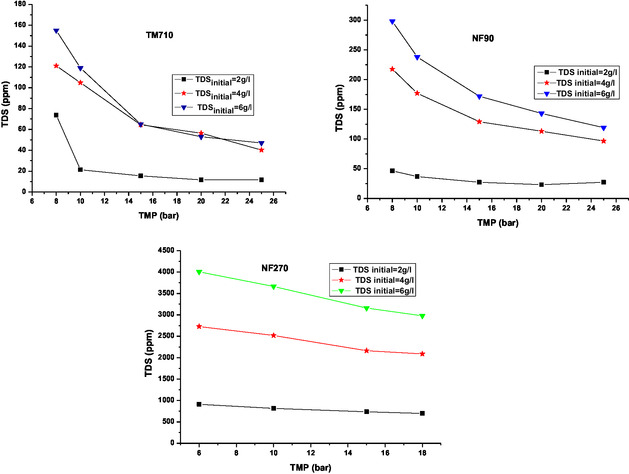
TDS permeate as a function of TMP for the membranes tested.

This section examines the effect of TMP on TDS for three BW samples, using a single‐pass configuration in continuous mode for the three membranes. **Figure** **4** illustrates the variation in permeate TDS as a function of the TMP.

Figure 4 shows that the permeate TDS has decreased as the TMP increases for the three membranes studied in the three feed TDS. It can be seen from this figure that TM710 membrane have higher TDS rejection rates than NF270 and NF90 membranes. However, the NF90 membrane shows TDS values which are close to those obtained by TM710.

### 4.3 Effects of TMP and a Feed TDS on Na^+^ and Cl^−^ Concentration in Permeate

**Figure 5:**
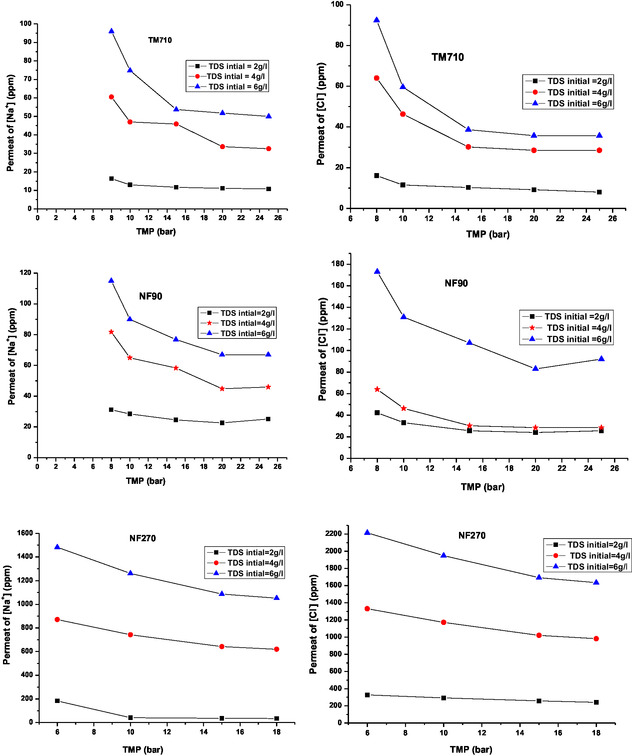
Evolution of Na^+^ and Cl^‐^ concentration in permeate with the TMP at three feed TDS.

The effect of applied pressure on the Na^+^ and Cl^−^ concentration in the permeate was studied for three BW samples, with three feed TDS levels, in a single pass configuration in continuous mode, using three membranes. **Figure** **5** illustrates the variation in Na+ and Cl‐ concentration in the permeate as a function of applied pressure, for the different feed TDS levels and the membranes examined.

For the three membranes tested, the concentration of Na^+^ and Cl^−^ ions in the permeate decreases with the TMP in the studied salinity range. This decrease is less pronounced for the NF270 membrane, which appears to be less influenced by the salinity effect compared to the other membranes tested. For a given TMP, the concentration of Na^+^ and Cl^−^ ions in the permeate increases with salinity, along with the simultaneous reduction in permeate flux. The NF270 membrane, with its more porous structure, offers better permeability but is less sensitive to salinity changes. In contrast, the denser NF90 and TM710 membranes are more resistant to ion diffusion, making them more selective and less influenced by salinity. This difference is linked to pore size and the level of Na^+^ and Cl^−^ ion rejection.^[^ ^40^ ^]^

### 4.4 Mathematical Models

#### 4.4.1 Kedem–Katchalsky Model Fitting

**Figure 6:**
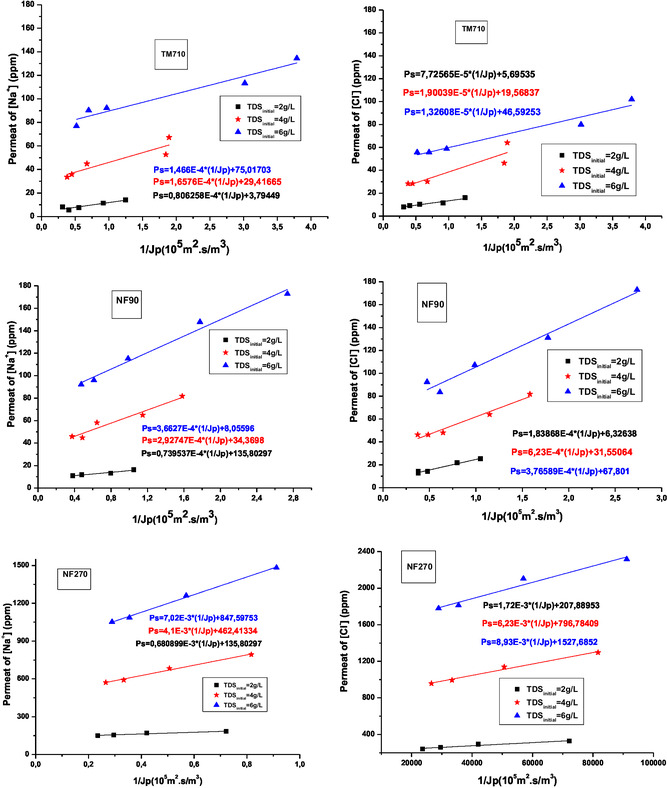
Evolution of permeate concentration of ions (Na^+^ and Cl^−^) as a function of the ratio 1/J_v_.

To separately quantify the two modes of solute mass transfer (convection and solution/diffusion) occurring in the tested membranes, the variation of C_p_ as a function of the inverse of the permeate flux is plotted according to (Equation 8). **Figure** **6** reveals a linear relationship C_p _= f(1/J_diff_), consistent with the equation.

The results allow for determining whether the transfer mechanism of sodium and chloride ions in each membrane is diffusive or convective, while also examining the influence of the initial TDS of the feed Water on these mechanisms.

The intersection point on the ordinate axis of the curve C_p _= f(1/J_diff_) gives the C_conv_ value, and the slope gives the value of J_diff_. Values of C_conv_ and J_diff_ for the three membranes are reported in **Table** **3**.

**Table 3:** C_conv_ and J_diff_ values obtained at different initial feed TDS.

Ions		Sodium Na^+^			Chloride Cl^−^		
Membrane	TDS [g L^−1^]	C_conv_ [g L^−1^]*10^−3^	J_diff_ [Kg m^−2 ^s^−1^]*10^−7^	R‐square	C_conv_ [g L^−1^]*10^−3^	J_diff_ [Kg m^−2 ^s^−1^]*10^−7^	R‐square
TM710	2	3.79449	0.80625	0.81	5.6535	0.772565	0.91
	4	29.41665	1.65761	0.79	19.56837	1.90039	0.79
	6	75.01703	1.466	0.92	46.59253	1.32608	0.93
NF90	2	8.05596	0.73954	0.92	6.32638	1.83868	0.96
	4	34.33698	2.92747	0.92	31.55064	3.04154	0.95
	6	76.54844	3.6627	0.97	67.801	3.76589	0.96
NF270	2	135.80297	6.80899	0.90	207.88953	17.20	0.91
	4	462.41334	41.00	0.98	796.78409	62.30	0.97
	6	847.59753	70.20	0.99	1527.6852	89.30	0.95

**Figure 7:**
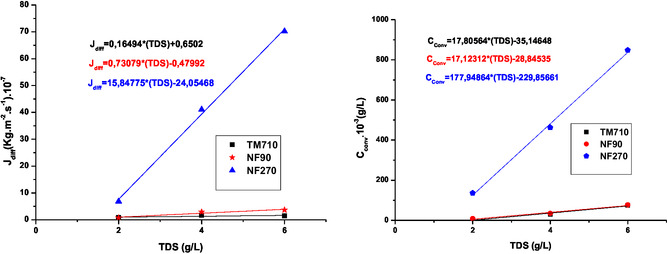
Variation of diffusion flux J_diff_ and convection concentration C_conv_ for sodium ion as a function of initial feed salinity.

**Figure 8:**
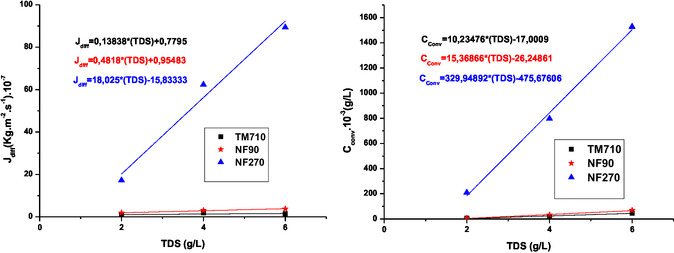
Variation of diffusion flux Jdiff and convection concentration C_conv_ for chloride ion as a function of initial feed salinity.

**Figure** **7** and **8** illustrate the variation of diffusion flux J_diff_ and convection concentration C_conv_ for sodium and chloride ions as a function of initial feed TDS.

The Figure 7 and 8 show that for NF270 membrane increasing feed water salinity increase sharply both diffusive transport (J_diff_) and convective transport (C_conv_). We can notice that NF270 is more convective compared to the NF90 membrane.^[^ ^31^ ^,^ ^38^ ^]^

The C_conv_ value of the TM710 membrane tends to zero, so this membrane is almost diffusive.^[^ ^38^ ^]^ Increasing TDS has no influence on the diffusion flux for both TM710 and NF90, while the C_conv_ increases but unsignificantly. This shows that the NF90 membrane has very similar mass transfer properties to the TM710 RO membrane. For NF270 both the diffusion flux and the convection concentration increase sharply with TDS. This can be attributed to the polarization concentration phenomena widely encountered in the crossflow NF process.^[^ ^39^ ^]^

#### 4.4.2 Spiegler—Kedem Model Fitting

**Figure 9:**
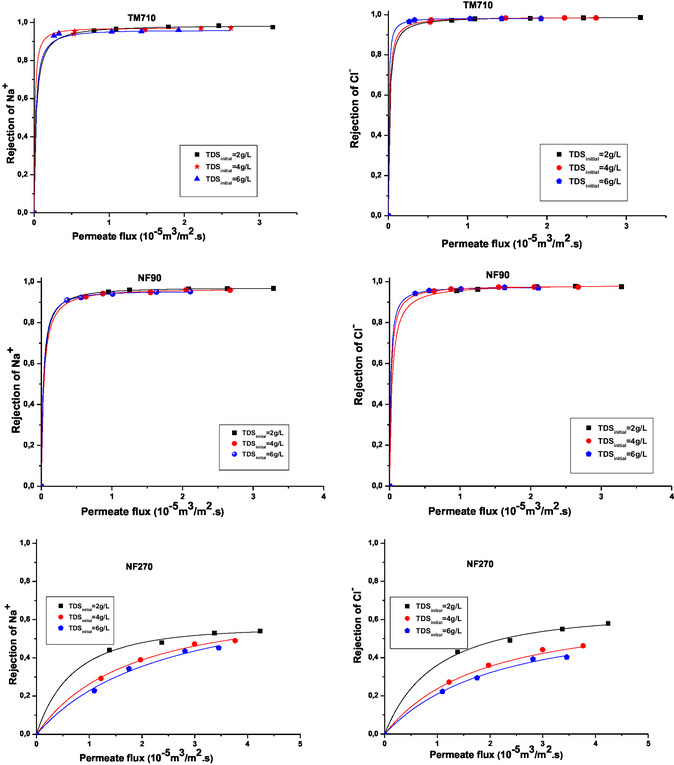
Experimental evolution of ions Na^+^ and Cl^−^ rejection with permeation flux for the three membranes in three initial TDS. The curves were fitted by the SK model.

The experimental data of rejection for the Na^+^ and Cl^−^ ions and permeate flux for the three tested membranes in the three initial feed TDS was fitted using the SK model to determine the salt permeability (P_s_) and reflection coefficient (*σ*) parameters. A good fit was obtained for the rejection values for the three membranes in the three feed TDS studied. This is shown in **Figure** **9**, which compares experimental values with calculated values using the best fit values of P_s_ and *σ* obtained by regression of data according to (Equation 12). Experimental data used are marked as solid symbols, whereas dash lines represent the SK model.

With regard to the reflection coefficient, the closer it is to unity, the more difficulty the solute will have in crossing the membrane. A value equal to zero designates that the membrane is fully permeable to the solute.^[^ ^32^ ^]^

The calculated rejections using the estimated *σ* and P_s_ values were shown in Figure 9. The *σ* and P_s_ values for Na^+^ and Cl^−^ ions obtained by fitting of experimental data using the SK model for each membrane studied in the three feed TDS are summarized in **Table** **4**. It is evident that the rejection increases with increasing permeate flux for the membranes tested. The results obtained in Table 4 show clearly that values of P_s_ and *σ* are dependent on the type of ions, the TDS feed, and also on the membrane used. The *σ* values obtained by both TM710 and NF90 membranes are in the three feed TDS close to 1, which means that these membranes are much closer to the ideal case that gives a complete rejection. This result means that the transfer of ions through these membranes is done largely by diffusion.^[^ ^38^ ^]^ The value obtained by NF270 membrane decreases due to the reduction of salt rejection. The P_s_ values of these membranes increase with TDS. The P_s_ of the NF270 membrane with the strong negative charge is more influenced by the high feed TDS than the NF90.^[^ ^33^ ^]^ On the other hand, the NF membranes have low values of *σ* for NaCl salt due to the lower rejection values of the monovalent salt. The NF270 which has a low rejection of NaCl presents the lower *σ* and the higher P_s_ values. A good agreement between the experimental and calculated rejection data was confirmed at three feed TDS.

**Table 4:** Calculated values of reflection coefficient (*σ*) and solute permeability (P_s_) for both ions (Na^+^and Cl^−^) of filtered brackish waters for the tested membranes at initial feed TDS.

Ions		Sodium Na^+^			Chloride Cl^−^		
Membrane	TDS [g L^−1^]	P_s_ [m^3 ^m^−2 ^s^−1^]*10^−7^	*σ*	R‐square	P_s_ [m^3 ^m^−2 ^s^−1^]*10^−7^	*σ*	R‐square
TM710	2	2.73424	0.9828	0.99	1.57808	0.98646	0.99
	4	2.16308	0.95618	0.99	1.24858	0.98525	0.99
	6	1.07417	0.96714	0.99	0.59389	0.98486	0.99
NF90	2	2.91404	0.96799	0.99	3.9687	0.9854	0.99
	4	3.34468	0.96044	0.99	1.84659	0.98214	0.99
	6	3.57322	0.9508	0.99	1.59317	0.97096	0.99
NF270	2	63.7462	0.54805	0.99	87.8248	0.61286	0.99
	4	156.721	0.62688	0.99	189.481	0.60663	0.99
	6	217.726	0.68932	0.99	158.008	0.58673	0.99

**Figure 10:**
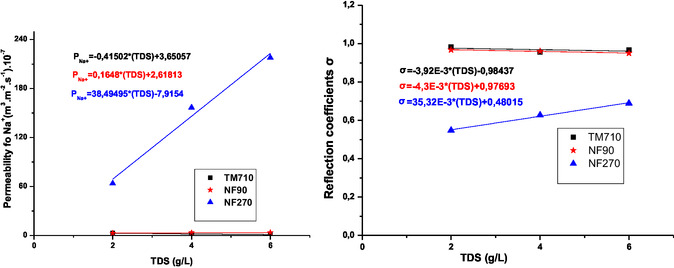
Variation of reflection coefficients (*σ*) and solutes permeabilities P_s_ for sodium ion as a function of feed TDS.

On the other hand, for the NF270 membrane, *σ* is very low in the system, which can be explained by the fact that the transfer of ions was largely by convection (**Figure** **10**).^[^ ^31^ ^,^ ^38^ ^]^

**Figure 11:**
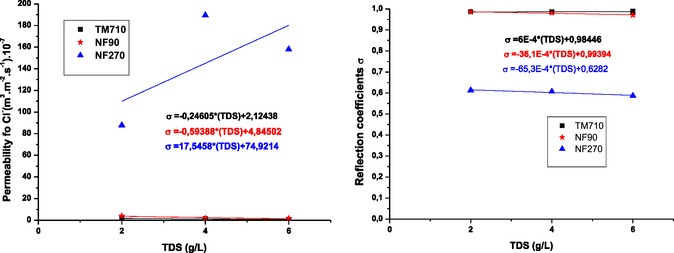
Variation of reflection coefficients (*σ*) and solutes permeabilities P_s_ for chloride ion as a function of feed TDS.

The result obtained in **Figure** **11** shows that the *σ* and P_s_ values for sodium and chloride ions obtained by NF90 and TM710 membrane are almost equal and almost independent of TDS of feed water. NF90 membrane has similar properties to TM710 membrane which is a RO membrane as previously mentioned.

For the NF270 membrane, the *σ* value for sodium ion increases with increasing feed water TDS, while it remains almost constant for chloride ion with increasing feed water TDS. The P_s_ values obtained for sodium and chloride ions increase with increasing feed water TDS, which can be attributed to to the decrease in retention rate with increasing feed water TDS.

## 5 Conclusion

This study examined the impact of salinity (2, 4, and 6 g L^‐1^) on the performance of two nanofiltration membranes (NF270 and NF90) and a reverse osmosis membrane (TM710), focusing on the transport mechanisms of sodium (Na^+^) and chloride (Cl^−^) ions. The results revealed distinct behaviors among the membranes: 1) NF270 exhibited the highest permeate flux but lower solute retention, with transport primarily driven by convection. As salinity increased, both its diffusive flux (Jdiff) and convective concentration (Cconv) rose. Additionally, higher total dissolved solids (TDS) concentrations led to a significant increase in solute permeability (Ps) and a decrease in the reflection coefficient (*σ*) for sodium, indicating reduced retention efficiency; 2) NF90 and TM710 displayed similar performance, predominantly governed by diffusion. Their reflection coefficient (*σ*) remained close to 1, and their low solute permeability (Ps) confirmed their strong ion retention capacity, even at higher salinities. Furthermore, the stability of *σ* and Ps across different TDS levels highlighted their robustness against salinity variations.

Overall, the NF270 membrane is well‐suited for applications requiring high permeate flow but with lower selectivity for monovalent ions. In contrast, the NF90 and TM710 membranes are better suited for applications demanding high ion rejection, such as the desalination of highly saline brackish water.

## Conflict of Interest

The authors declare no conflict of interest.

## Author Contributions

**A. Lachheb**: data curation: (lead); formal analysis: (equal), **F. Z. Addar**: writing—original draft: (equal); writing—review and editing: (supporting), **I. Kouda**: project administration: (lead); resources: (lead), **N. Zouhri**: formal analysis: (equal): **J. Touir** investigation: (equal), **M. Taky**: supervision: (supporting), **M. Tahaikt**: writing—original draft: (supporting); writing—review and editing: (supporting).

## Data Availability

The data that support the findings of this study are available from the corresponding author upon reasonable request.

## References

[1] J. R. Du , X. Zhang , X. Feng , Y. Wu , F. Cheng , M. E. A. Ali , Desalination 2020, 491, 114445.

[2] B. Mukhopadhyay , B. K. Mukhopadhyay , in The Sentinel 29th India 2020.

[3] W. Musie , Heliyon 2023, 9, e18685, 37554830 10.1016/j.heliyon.2023.e18685 PMC10405016

[4] A. Boretti , L. Rosa , npj Clean Water. 2019, 2, 15.

[5] P. K. Pandey , P. H. Kass , M. L. Soupir , S. Biswas , V. P. Singh , Amb Express 2014, 4, 51. 25006540 10.1186/s13568-014-0051-x PMC4077002

[6] D. Baudreu , Archéologie du Midi Médiéval 2014, 32, 233.

[7] A. D. Khawaji , I. K. Kutubkhanah , J.‐M. Wie , Desalination 2008, 221, 47.

[8] L. F. Greenlee , D. F. Lawler , B. D. Freeman , B. Marrot , P. Moulin , Water Res. 2009, 43, 2317. 19371922 10.1016/j.watres.2009.03.010

[9] S. M. Z. Javaid , F. Fadhillahb , Z. Khanc , A. F. Ismail , Desalination 2015, 368, 202.

[10] V. G. Molina , M. A. Marzal , K.‐U. Hoehn , Designing Membrane Systems for the Coming Future: Perth II Desalination Plant, IDA World Congress—Atlantis, UAE, The Palm‐Dubai 2009.

[11] K. Majamaa , F. Creus , A. Roy , J. Johnson , Economical Benefits of Extra Fouling Resistant Low Energy Reverse Osmosis Membranes in Municipal Wastewater Reuse Applications, International Desalination Association World Congress Perth, Australia 2011.

[12] E. Gasia‐Bruch , P. Sehn , V. García‐Molina , M. Busch , O. Raize , M. Negrin , Desalin. Water Treat. 2011, 31, 178.

[13] A. Saadi , N. Ouazzani , Desalination 2004, 165, 81.

[14] I. Munoz , A. R. Fernandez‐Alba , Water Res. 2015, 42, 801. 10.1016/j.watres.2007.08.021 17826817

[15] A. Lachheb , S. Belhamidi , N. Zouhri , Y. A. Idrissi , M. Hafsi , M. Taky , M. E. Amrani , A. Elmidaoui , J. Chem. Pharm. Res. 2016, 8, 119.

[16] N. Zouhri , M. L. Mohamed Igouzal , M. Hafsi , M. Taky , A. Elmidaoui , Desalin. Water Treat. 2018, 120, 41.

[17] H. Boulahfa , S. Belhamidi , F. Elhannouni , M. Taky , A. E. Fadilb , A. Elmidaoui , J. Environ. Chem. Eng. 2019, 7, 102937.

[18] A. Achilli , T. Y. Cath , E. A. Marchand , A. E. Childress , Desalination 2009, 239, 10.

[19] C. A. Yanu , N. Nguiamba , J. M. Sieliechi , M. B. Ngas‐soum J. Mater. Sci. Chem. Eng. 2023, 11, 43.

[20] D. Oatley‐Radcliffe , M. Walters , T. J. Ainscough , P. M. Williams , A. W. Mohammad , N. Hilal , J. Water Process Eng. 2017, 19, 164.

[21] M. Pontié , H. Dach , J. Leparc , M. Hafsi , A. Lhassani , Desalination 2008, 221, 174.

[22] M. Tahaikt , A. AitHaddou , R. E. Habbani , Z. Amor , F. E. hannouni , M. Taky , M. Kharif , A. Boughriba , M. Hafsi , A. Elmidaoui , Desalination 2008, 225, 209.

[23] M. Tahaikt , R. E. Habbani , A. AitHaddou , I. Achary , Z. Amor , M. Taky , A. Alami , A. Boughriba , M. Hafsi , A. Elmidaoui , Desalination 2007, 212, 46.

[24] N. Hilal , H. Al‐Zoubi , A. Mohammad , N. Darwish , Desalination 2005, 184, 315.

[25] M. F. Pouet , A. Grasmick , F. Homer , F. Nauleau , J. C. Cornier , Water Sci. Technol. 1994, 30, 133.

[26] S. Bandini , J. Membr. Sci. 2005, 264, 75.

[27] S. Bouranene , P. Fievet , A. Szymczyk , Chem. Eng. Sci. 2009, 64, 3789.

[28] C. Bartels , R. Franks , S. Rybar , M. Schierach , M. Wilf , Desalination 2005, 184, 185.

[29] J. Straatsma , G. Bargeman , H. C. van der Horst , J. A. Wesselingh , J. Membr. Sci. 2002, 198, 273.

[30] N. Hilal , H. Al‐Zoubi , N. A. Darwish , A. W. Mohammad , M. A. Arabi , Desalination 2004, 170, 281.

[31] M. Tahaikt , F. Elazhar , I. Mohamed , H. Zeggar , M. Taky , A. Elmidaoui , Desalin. Water Treat. 2021, 240, 14.

[32] A. M. Hidalgo , M. Gómez , M. D. Murcia , E. Gómez , G. León , A. Sánchez , Sep. Sci. Technol. 2016, 51, 2429.

[33] H. Al‐Zoubi , W. Omar , Korean J. Chem. Eng. 2009, 26, 799.

[34] Y. Zhang , H. Qian , K. Hou , W. Qu , Eng. Geol. 2021, 285, 106050.

[35] N. Zouhri , F. Z. Addar , M. Tahaikt , M. Elamrani , A. ELmidaoui , M. Taky , Desalin. Water Treat. 2024, 317, 100042.

[36] M. Tahaikt , S. El‐Ghzizel , N. Essafi , M. Hafsi , M. Taky , A. Elmidaoui , Desalin. Water Treat. 2021, 216, 83.

[37] A. Jeihanipour , J. Shen , G. Abbt‐Braun , S. A. Huber , G. Mkongo , A. I. Schäfer , Sci. Total Environ. 2018, 637, 1209. 29801214 10.1016/j.scitotenv.2018.05.113

[38] N. Zouhri , M. Igouzal , M. Larif , M. Hafsi , M. Taky , A. Elmidaoui , Desalin. Water Treat. 2018, 120, 41.

[39] Y. Zhang , L. Zhang , L. Hou , S. Kuang , A. Yu , AIChE J. 2019, 65, 1076.

[40] M. Tahaikt , F. Elazhar , I. Mohamed , H. Zeggar , M. Taky , Desalin. Water Treat. 2021, 240, 14.

[41] G. Artuğ , I. Roosmasari , K. Richau , J. Hapke , Sep. Sci. Technol. 2007, 42, 2947

